# Triethylamine-Capped Calcium Phosphate Oligomers/Polyacrylamide Synergistically Reinforced *α*-Hemihydrate Gypsum Composites: A Mechanistic Study on Mechanical Strengthening via Organic/Inorganic Interpenetrating Networks

**DOI:** 10.3390/molecules30092002

**Published:** 2025-04-30

**Authors:** Yuan Chen, Li Chen, Hao Li, Bin Zhang, Marie-Christine Record, Pascal Boulet, Juan Wang, Jan-Michael Albina, Yi Yang, Weiliang Ma

**Affiliations:** 1 Hubei Provincial Key Laboratory of Green Materials for Light Industry, Collaborative Innovation Center of Green Light-Weight Materials and Processing, School of Materials and Chemical Engineering, Hubei University of Technology, Wuhan 430068, China; chen.yuan@hbut.edu.cn (Y.C.); 102410475@hbut.edu.cn (B.Z.); jwang@hbut.edu.cn (J.W.); albina@hbut.edu.cn (J.-M.A.); yangyi@hbut.edu.cn (Y.Y.); 2 New Materials and Green Manufacturing Talent Introduction and Innovation Demonstration Base, Wuhan 430068, China; m-c.record@univ-amu.fr (M.-C.R.); pascal.boulet@univ-amu.fr (P.B.); 3 Campus Saint-Jérôme, Aix-Marseille University, 13397 Marseille, CEDEX 20, France; 4 Institut Matériaux Microélectronique Nanosciences de Provence (IM2NP), 13397 Marseille, CEDEX 20, France

**Keywords:** *α*-type hemihydrate gypsum, gypsum polymer composites, polyacrylamide hydrogel, multi-network structure, mechanical enhancement, gypsum modification

## Abstract

In this study, a novel calcium phosphate/polyacrylamide copolymer/α-type hemihydrate gypsum (CPO/PAM/α-HHG) composite material was prepared by polymerising a stable inorganic CPO precursor, end-capped with triethylamine (TEA), with an organic polyacrylamide (PAM) hydrogel to form a CPO/PAM precursor solution. Subsequently, this precursor solution was mixed with inorganic α-hemihydrate gypsum. The effects of CPO/PAM precursor addition and CPO addition on the slurry flowability, initial setting time, and mechanical properties of hardened specimens of the CPO/PAM/α-HHG composite were investigated. The structural characteristics of the composites were analysed by XRD, FE-SEM, and TGA. The results show that the initial setting time of the CPO/PAM/α-HHG composites was 26.7 min, which was 140.5% longer than that of the pure water α-HHG system and 3.9% longer than that of the PAM/α-HHG system; additionally, the oven-dried specimens had a flexural strength of 27.59 MPa and a compressive strength of 68.48 MPa, which were 77.2% and 102.0% higher than those of the pure water α-HHG system and 38.8% and 14.1% higher than those of the PAM/α-HHG system, respectively. The wet compressive strength of the CPO/PAM/α-HHG composites was improved by 11.8% compared to that of the PAM/α-HHG system. A structural analysis showed that CPO promoted the gelation process of PAM and allowed the hydration reaction process of α-HHG to be fully carried out by slowing down the gelation process of the organic network, which led to the full development of both organic and inorganic networks, ultimately forming an interspersed inorganic/organic dual-network structure, which enhanced the comprehensive mechanical properties of the composites. This study provides a new idea for the modification of α-type hemihydrate gypsum and a new method for the preparation of high-utilisation and high-performance gypsum-based composites.

## 1. Introduction

Gypsum is a high-quality building material known for its lightweight, fire resistance, sound insulation, and thermal insulation properties. It also boasts a simple production process with low energy consumption, making it an ideal choice for efficient, green, and energy-saving construction materials [1,2,3,4]. Gypsum can be categorised into dihydrate gypsum, hemihydrate gypsum (α-type and β-type), and anhydrous gypsum (Types I, II, and III). Among these, α-type hemihydrate gypsum (α-HHG) is distinguished by its large crystal grains and low specific surface area [5], enabling it to rapidly form a uniform slurry upon hydration, which gradually hardens over time into a high-strength and densely structured solid [6,7]. However, α-HHG has some drawbacks: its hydration and hardening rate is fast, which may cause time pressure on construction and formation; in addition, the mechanical strength of traditional α-HHG has difficulties in meeting the needs of practical engineering development [8].

Polymer hydrogels are cross-linked polymers that significantly swell in water, retaining a substantial amount of moisture without dissolving [9,10]. Polyacrylamide (PAM) hydrogels represent a major category of synthetic polymer hydrogels, characterised by a straightforward synthesis process and a structure rich in amide groups [11,12]. These amide groups readily interact to form hydrogen bonds, which confer an excellent water absorption capacity and stability to the hydrogels [13]. In recent years, a significant amount of literature has reported on modification studies of polyacrylamide to enhance its mechanical properties [14,15,16,17,18]. Yu et al. [19] used an organic acrylamide (AM) monomer and triethylamine (TEA)-capped inorganic calcium phosphate (CPO) oligomers as precursors to prepare a structurally uniform calcium phosphate/polyacrylamide copolymer (CPO/PAM) via an organic/inorganic copolymerisation method. The mechanical properties of this copolymer were found to be significantly superior to those of traditional PAM.

To adjust the initial setting time of gypsum materials and improve their mechanical properties, many researchers have carried out modification studies. For example, physical modifications have been made to alter the morphology and size of gypsum particles, enhancing their overall performance [20,21,22]. Alternatively, chemical modifications involve the addition of organic or inorganic additives to gypsum materials, which optimise their chemical composition and microstructure, thereby improving the microstructure of hydration and hardening products, as well as the overall performance [23,24,25,26,27]. Patrycja Mroz et al. [28] utilised hydroxyethyl methylcellulose as an additive to modify the crystal morphology of dihydrate calcium sulphate, enhancing the bending resistance of the material. Thompson et al. [29] employed hydrogel beads and methylcellulose to regulate the templating of gypsum slurry, adjusting the porosity and pore size distribution of the gypsum material, which improved its water resistance and sound insulation properties. Mi et al. [30] developed a PAM/α-HHG composite material by combining organic PAM with inorganic α-HHG. This composite displayed a significantly longer initial setting time than traditional α-HHG while also achieving notable enhancements in mechanical properties.

Based on the aforementioned approach, this study prepared a CPO/PAM precursor solution and combined it with α-HHG to ultimately obtain a CPO/PAM/α-HHG composite material. By introducing CPO, the density of the organic PAM network was effectively enhanced, while the hydration degree of α-HHG was improved, thus enhancing the mechanical properties of the PAM/α-HHG composite material. This efficient and streamlined approach not only provides a novel avenue and methodology for the modification of α-HHG but also aligns with sustainable development goals, paving the way for the practical application of high-performance α-HHG materials.

## 2. Results and Discussion

### 2.1. Influence of CPO/PAM Precursor Addition on the Properties of CPO/PAM/α-HHG Composites

#### 2.1.1. Influence on the Flowability of CPO/PAM/α-HHG Composite Slurries

**Figure 1:**
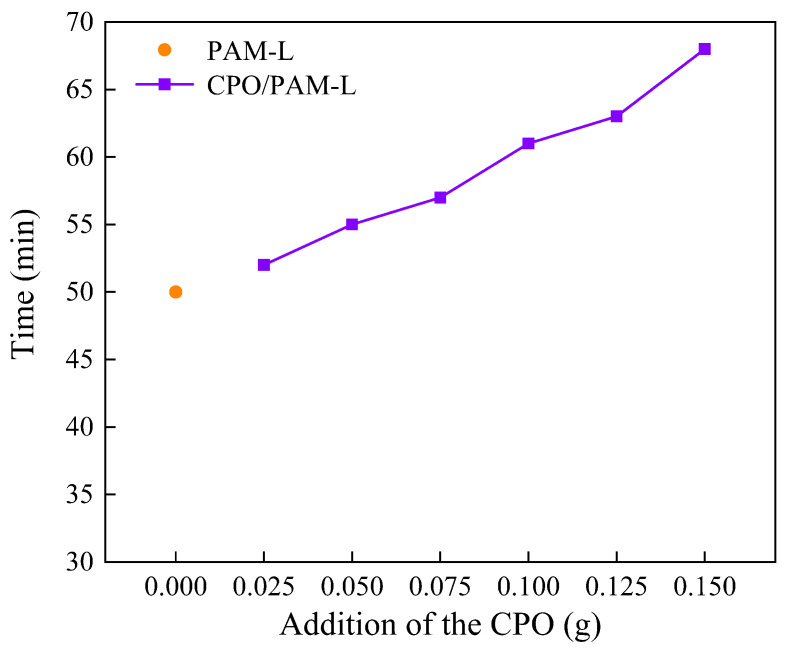
Curves of the drawing time of the CPO/PAM hydrogel with the addition of CPO.

In this study, the polymerisation time of CPO/PAM composite hydrogels is characterised by the drawable time, as illustrated in Figure 1. The results indicate that the introduction of CPO significantly extends the drawable time of the composite gel, with it reaching 61 min at a CPO addition of 0.1 g, which is 22% longer than that of the PAM control group without added CPO (50 min). The hydrolysis of phosphate ions in CPO releases OH^−^, resulting in an increase in the pH value of system. This alkaline environment inhibits the decomposition process of ammonium persulphate (APS) and reduces the free radical generation rate, thereby prolonging the polymerisation kinetics of the hydrogel. Furthermore, the polymerisation of multiple components within the system increases the overall reaction time, contributing to a further extension of the drawable time.

**Figure 2:**
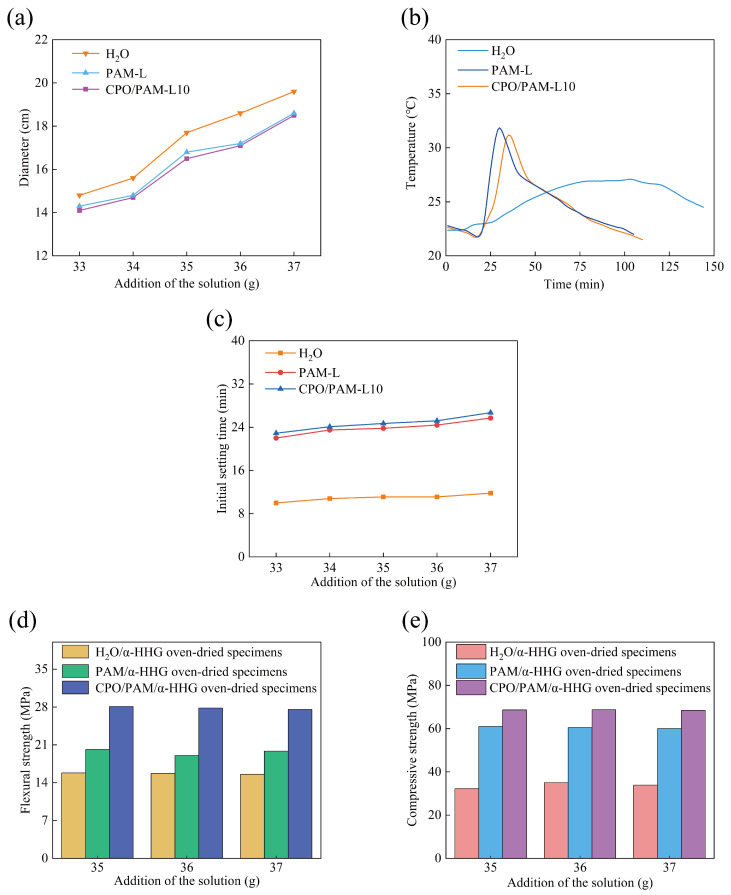
Variation in the properties of the CPO/PAM/α-HHG composite material with the addition of CPO/PAM precursor solution: (**a**) flowability, (**b**) hydration–hardening temperature, (**c**) initial setting time, (**d**) flexural strength, and (**e**) compressive strength. The horizontal axis, labelled “Addition of the solution”, indicates the mass of solution added per 100 g of α-HHG.

Aiming to meet the operability requirements of gypsum-based materials in practical applications, this study focuses on the effect of CPO/PAM precursor liquid addition on the flowability of CPO/PAM/α-HHG composite slurry. The effect of precursor liquid addition on the flowability of composite pastes is illustrated in Figure 2a. The results indicate that, when the addition amounts are all 36 g (the water requirement for the standard consistency of α-HHG is 36 g), the flowability of the PAM/α-HHG system decreases slightly (2.7%) compared to that of the pure water control group. This decrease is attributed to the increase in viscosity due to the high hydrophilicity of the PAM network. The CPO/PAM-L10 group (17.1 cm) demonstrates comparable flowability to the PAM-L group (17.2 cm), indicating that the short-term polymerisation reaction between CPO and PAM does not have a significant effect on the flowability of the composite pastes. The composite slurry consistency is closest to the standard consistency of α-HHG when the CPO/PAM-L10 precursor liquid addition is 37 g.

#### 2.1.2. Influence on the Hydration–Hardening Temperature of CPO/PAM/α-HHG Composites

The temperature variation patterns of the α-HHG hydration and hardening processes with different types of precursor liquids (36 g for the pure water group and 37 g for the PAM-L and CPO/PAM-L10 groups) are illustrated in Figure 2b. In the pure water system, the peak hydration temperature of α-HHG occurs at 103 min (27 °C), and the temperature of the system increases slowly. In contrast, the PAM-L solution exhibits a typical three-phase exothermic characteristic: an initial temperature drop, a rapid warming period (which occurs significantly faster than that of the pure water control group, reaching a peak temperature of 31.85 °C at 30 min), and a slow cooling period. The rapid increase in temperature during the warming period results from the exothermic PAM gelation reaction, a phenomenon reported in our previous work [30]. A comparison between the pure water group and the PAM-L group reveals that the presence of α-HHG retards the gelation reaction of the AM solution. Since water activity is one of the key factors controlling the rate of PAM gelation, and water is necessary for both PAM gelation and α-HHG hydration [7,31,32,33], this phenomenon implies that, in the α-HHG/PAM system, the hydration of inorganic α-HHG and the gelation of the organic PAM solution may compete for water. This competition for water retards both reactions.

The temperature change curve of the CPO/PAM-L10 group is similar to that of the PAM-L group, indicating that the CPO/PAM network maintains its hydrogel properties. The curve reaches a peak temperature of 31.17 °C at 35 min, with the time to peak delayed by 5 min compared to that of the PAM-L group. This delay is attributed to the introduction of CPO, which prolongs the gelation process of PAM, and this is corroborated by the results on the drawable time in Section 2.1.1.

#### 2.1.3. Influence on the Initial Setting Time of CPO/PAM/α-HHG Composites

The effects of different additions of precursor liquid on the initial setting time of the CPO/PAM-L10 composites are illustrated in Figure 2c. Compared to the pure water control group (11.8 min, at standard consistency), the initial setting time of the CPO/PAM/α-HHG system (with 37 g of precursor liquid addition) is significantly extended to 26.75 min (an increase of 111.0%). Additionally, when compared to the PAM-L group (25.75 min, with 37 g of precursor liquid addition), the initial solidification time of the CPO/PAM-L10 composites is extended by 3.9%. The CPO/PAM-L10 composites demonstrate a positive correlation between the initial setting time and the amount of precursor liquid added, reaching the maximum value when 37 g is added. Detailed data are provided in Table S1.

The extended initial setting time of the CPO/PAM/α-HHG system can be attributed to two mechanisms: On the one hand, the hydration hardening process of α-HHG competes with the gelation reaction of PAM for water availability. On the other hand, the introduction of CPO prolongs the gelation time of PAM, further delaying the formation of the organic network and slowing down the overall reaction process of the organic/inorganic composite system. Consequently, this results in an extended initial setting time for the composite slurry. In practical engineering applications, the significant prolongation of the initial setting time enhances the flow, shaping, and operability of the CPO/PAM/α-HHG composite material slurry, which holds considerable positive significance for practical engineering.

#### 2.1.4. Influence on the Mechanical Properties of CPO/PAM/α-HHG Composites

Figure 2d,e demonstrate the flexural and compressive strengths of oven-dried CPO/PAM/α-HHG-L10 composite specimens. At a precursor addition of 37 g, the CPO/PAM-L10 system exhibits remarkable improvements in mechanical properties. The flexural strength reaches 27.59 MPa, representing increases of 77.2% compared to the pure water group (15.57 MPa) and 38.8% compared to the PAM-L group (19.87 MPa). Similarly, the compressive strength increases to 68.48 MPa, marking improvements of 102.0% and 14.1% compared with the pure water group (33.90 MPa) and the PAM-L group (60.08 MPa), respectively. Detailed data of the mechanical properties are shown in Tables S2 and S3.

The enhanced mechanical properties are attributed to the combined action of two mechanisms. Firstly, the incorporation of CPO effectively prolongs the initial solidification period, providing sufficient time for the hydration reaction of α-HHG and the gelation of PAM to fully proceed. Consequently, both inorganic and organic phases develop thoroughly, creating an integrated inorganic/organic dual-network structure. Secondly, due to the capping effect of TEA, nanoscale CPO structures are formed, whose phosphate groups efficiently cross-link with the NH_2_ groups of PAM chains via hydrogen bonds, resulting in a more densely packed organic network.

Overall, under conditions of standard consistency, the optimised CPO/PAM-L10 composite exhibits significant enhancement in both the initial setting time (26.7 min) and mechanical properties (flexural strength: 27.59 MPa; compressive strength: 68.48 MPa). Notably, the compressive strength exceeds the conventional performance threshold for gypsum-based composites (65.00 MPa). This considerable improvement in mechanical properties is primarily due to the formation of a fully developed and highly interconnected inorganic/organic dual network.

### 2.2. Influence of CPO’s Addition in Precursor Solution on the Properties of CPO/PAM/α-HHG Composites

In this section, the effect of CPO addition on the properties of CPO/PAM/α-HHG composites is investigated. Based on the results in Section 2.1.3, the addition mass of CPO/PAM precursor solution is determined to be 37 g (per 100 g of α-HHG) to ensure that the consistency of the CPO/PAM/α-HHG composite slurry is close to the standard consistency of α-HHG.

#### 2.2.1. Influence on the Flowability of CPO/PAM/α-HHG Composite Slurries

**Figure 3:**
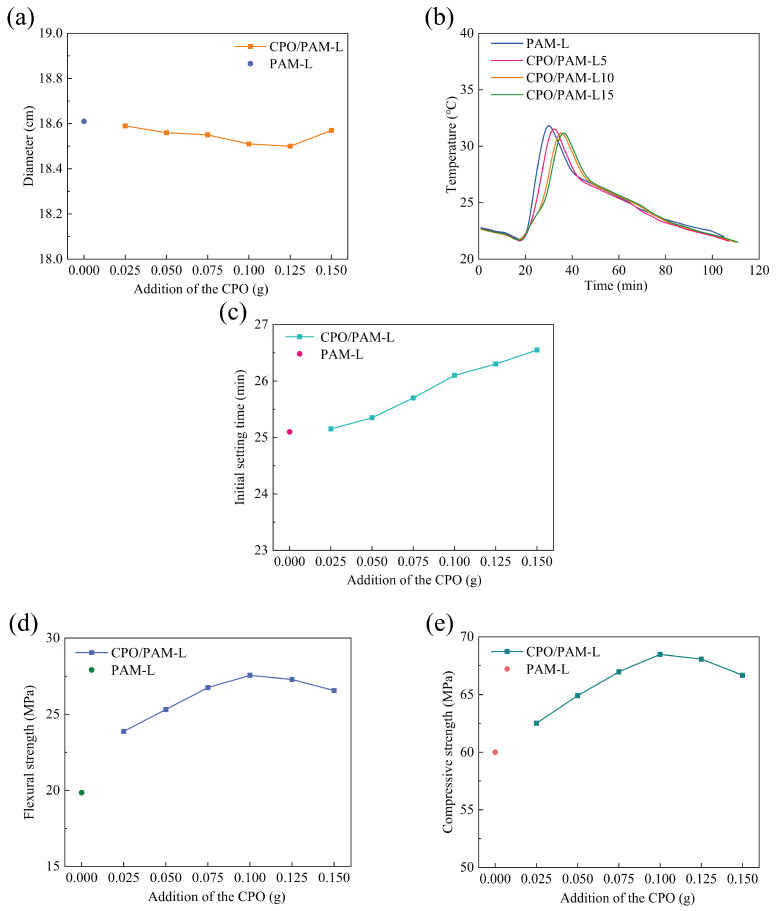
Variation in the properties of the CPO/PAM/α-HHG composite material with the amount of CPO added to the precursor solution: (**a**) flowability, (**b**) hydration–hardening temperature, (**c**) initial setting time, (**d**) flexural strength, and (**e**) compressive strength. The horizontal axis, labelled “Addition of the CPO”, indicates the amount of CPO added to the precursor solution per serving.

Figure 3a demonstrates the effect of CPO addition on the flowability of the CPO/PAM/α-HHG composite slurry. The flowability of the PAM-L group composite slurry without CPO addition, serving as the control group, is 18.6 cm. The flowability of the CPO/PAM/α-HHG composite slurry decreases slightly with increasing CPO addition; however, the flowability fluctuations remain within the range of 18.5–18.6 cm, with the maximum difference being less than 0.6%. The results are consistent with the conclusions of the rheological dynamics analysis in Section 2.1.1, indicating that the interaction effect between CPO and PAM within a relatively short period is very limited, and this effect does not significantly influence the flowability of the composite slurry.

#### 2.2.2. Influence on the Hydration–Hardening Temperature of CPO/PAM/α-HHG Composites

Figure 3b illustrates the influence of varying CPO addition levels (CPO/PAM-L5, CPO/PAM-L10, and CPO/PAM-L15) on the hydration and hardening temperatures of the CPO/PAM/α-HHG composites. The control group (PAM-L) reaches its maximum temperature (31.85 °C) at 30 min. In contrast, the CPO/PAM-L5, CPO/PAM-L10, and CPO/PAM-L15 groups achieve their peak temperatures of 31.67 °C, 31.17 °C, and 31.15 °C at 32 min, 35 min, and 36 min, respectively. The maximum temperature variation among these groups is approximately 0.6 °C. These observations demonstrate that adding CPO effectively slows down the hydration reaction in the PAM/α-HHG system, and this delay becomes more pronounced with a higher CPO content. Additionally, the peak hydration temperatures consistently decrease as the amount of CPO increases, primarily due to the inhibition effect of CPO on the gelation of PAM (see Section 2.1.1).

#### 2.2.3. Influence on the Initial Setting Time of CPO/PAM/α-HHG Composites

The effect of CPO addition on the initial setting time of the CPO/PAM/α-HHG system is shown in Figure 3c. The initial setting time of the PAM-L control group is 25.08 min, while the initial setting times of the CPO/PAM-L2.5, CPO/PAM-L5, CPO/PAM-L7.5, CPO/PAM-L10, CPO/PAM-L12.5, and CPO/PAM-L15 groups are 25.17, 25.33, 25.75, 26.08, 26.33, and 26.5 min, respectively, demonstrating a significant positive correlation with the additions. Compared to the PAM-L control group, the initial setting time is prolonged in all experimental groups, with the CPO/PAM-L15 group showing the greatest prolongation (about 6.0%). These data confirm that the introduction of CPO effectively prolongs the initial setting time of the composites, further optimising the operability of the composite pastes in practical engineering applications. Detailed data of the initial setting time are shown in Table S4.

#### 2.2.4. Influence on the Mechanical Properties of CPO/PAM/α-HHG Composites

**Figure 4:**
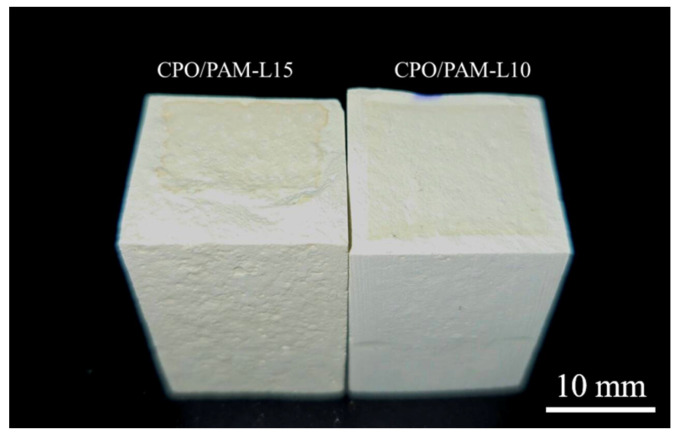
Disordered distribution of the gel network within the composite material.

Figure 3d illustrates the trend of the flexural strength of oven-dried specimens of the CPO/PAM/α-HHG composites with CPO addition. The figure shows that the flexural strength of the CPO/PAM/α-HHG system first increases and then decreases with increasing CPO addition. At an addition level of 0.1 g CPO, the composite flexural strength reaches a peak of 27.56 MPa, which is 38.8% higher than that of the PAM-L control. This is due to the polymerisation reaction between CPO and AM, which results in a more complete development of the organic gel network, effectively enhancing the densities of this network. The interpenetration of the organic and inorganic networks also improves the mechanical properties of the composites. However, further increasing the amount of CPO leads to a decrease in the flexural strength of the composites. The flexural strength of the CPO/PAM-L15 specimen is 26.56 MPa (a 3.6% decrease compared to the peak strength) when the addition of CPO reaches 0.15 g. At a macroscopic level, the CPO/PAM-L15 specimen exhibits a disordered distribution of the organogel network within the gypsum matrix (as shown in Figure 4). This phenomenon occurs because excessive CPO may raise the pH value of the precursor solution, negatively impacting the degree of polymerisation of the organogel. This disruption affects the uniformity of the organogel network distribution within the system, consequently destroying the interfacial synergy of the dual network [34].

The curves of the compressive strength of the oven-dried specimens of the CPO/PAM/α-HHG composites with CPO addition are presented in Figure 3e. The compressive strength and flexural strength of the composites exhibit similar trends. The oven-dried specimens achieve a peak compressive strength of 68.48 MPa with the addition of 0.1 g of CPO, which is 14.1% higher than that of the PAM-L control.

Combining the results of Section 2.1 and Section 2.2, the CPO/PAM precursor liquid achieves peak enhancement of the mechanical properties of α-HHG when CPO is added at a level of 0.1 g, with the flexural and compressive strengths increasing by 38.8% and 14.1%, respectively, in the oven-dried state compared to those of the PAM-L control. The composite system maintains fluidity (with a fluidity deviation of less than 0.6%) while significantly enhancing the mechanical properties through the synergistic enhancement mechanism of the organic/inorganic network, and it extends the operating time of the composite slurry by about 4.0%. The low cost of synthesis of the CPO/PAM precursor liquid and the simple production method, which demonstrates the characteristics of “low cost–high gain”, make CPO/PAM/α-HHG composites suitable for building fabrication and geological reinforcement, providing new ideas for the high-performance reinforcement of gypsum-based materials.

### 2.3. Phase Analysis of CPO/PAM/α-HHG Composite Materials

**Figure 5:**
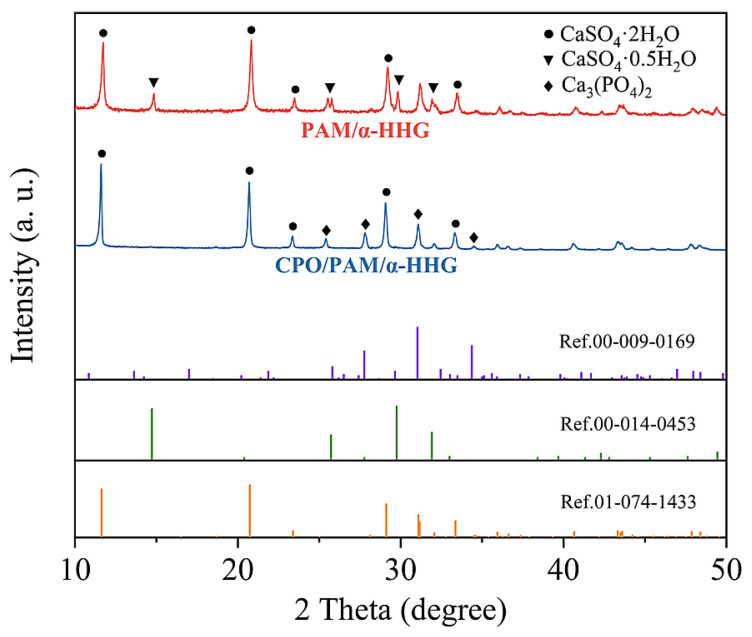
X-ray diffraction patterns of the CPO/PAM/α-HHG (CPO/PAM-L10) composite material and PAM/α-HHG composite material in oven-dried states.

The X-ray diffraction patterns of the hydration–hardened oven-dried specimens of the PAM/α-HHG composites and CPO/PAM/α-HHG composites (the CPO/PAM-L10 group) are presented in Figure 5. The red curve corresponds to the X-ray diffraction pattern of the oven-dried specimens of PAM/α-HHG, exhibiting characteristic diffraction peaks for gypsum dihydrate (CaSO_4_·2H_2_O, Ref. 01-074-1433) and α-gypsum hemihydrate (CaSO_4_·0.5H_2_O, Ref. 00-014-0453). This indicates that unreacted α-hemihydrate gypsum is present within the composite alongside the gypsum dihydrate product. The presence of residual hemihydrate gypsum results from the competition between the hydration process of α-HHG and the gelation process of PAM regarding water consumption. The faster development of the PAM hydrogel network depletes water in the system, preventing some α-HHG from reacting adequately with the water, as discussed in detail in our previous work [30]. The blue curve represents the X-ray diffraction pattern of the CPO/PAM/α-HHG oven-dried specimens, showing characteristic diffraction peaks for gypsum dihydrate (CaSO_4_·2H_2_O, Ref. 01-074-1433) and calcium phosphate (Ca_3_(PO_4_)_2_, Ref. 00-009-0169). Interestingly, this result indicates that there is no residual α-hemihydrate gypsum phase in the CPO/PAM/α-HHG specimens. This suggests that the addition of CPO effectively prolongs the overall reaction time of the system, allowing for the full development of both organic and inorganic networks during the hydration–hardening process of the CPO/PAM/α-HHG composites. This enhancement increases the degree of bonding and densification between the two networks, subsequently improving the mechanical properties of the CPO/PAM/α-HHG composites, which confirms the results presented in Section 2.1.3 and Section 2.2.3.

### 2.4. Fracture Analysis of CPO/PAM/α-HHG Composite Materials and Microstructural Analysis of TEA-Capped CPO

**Figure 6:**
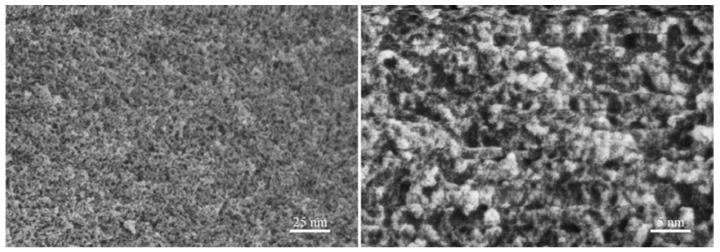
The TEA-stabilised inorganic CPO precursors.

Figure 6 presents FE-SEM photographs of the specimens obtained from the CPO gels after drying and milling. The images indicate that CPO polymerises upon the evaporation of TEA, forming clusters, all of which are below 10 nm in size, with some clusters being below 5 nm, consistent with the results reported in the literature [19]. This suggests that the size of the clusters in the CPO gels before drying may be smaller than that in the dry state, as described above. This implies that TEA exerts an effective capping effect on CPO, refining the size of the clusters in the CPO gels. On the one hand, CPO retards the gelation rate of the organic network in the CPO/PAM/α-HHG system; on the other hand, the very small CPO can effectively polymerise with PAM, facilitating the development of the gel network and substantially enhancing the densification of the organic network.

**Figure 7:**
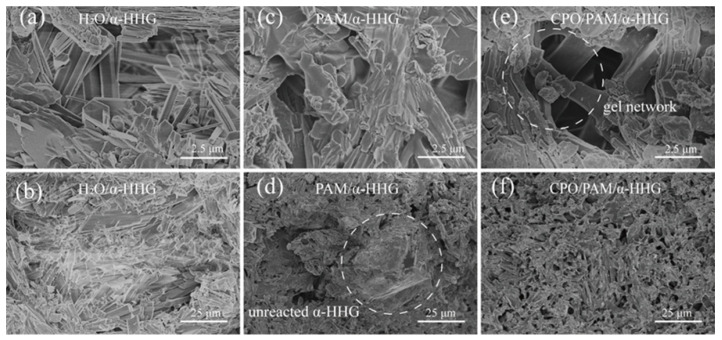
FE-SEM images of (**a**,**b**) oven-dried specimen fractures of H_2_O/α-HHG, (**c**,**d**) oven-dried specimen fractures of PAM/α-HHG, and (**e**,**f**) oven-dried specimen fractures of CPO/PAM/α-HHG.

Figure 7a,b present FE-SEM images of the fracture section of the oven-dried specimens from the pure water group (α-HHG/H_2_O). In the figures, it can be observed that the crystal surface at the fracture of the pure water group is smooth, the grain boundaries are distinct, and the crystals exhibit an elongated needle-like or lamellar structure with a small amount of fine crystal attachment. Figure 7c,d present FE-SEM images of the fracture section of the oven-dried specimens of PAM/α-HHG. The internal structure consists of a PAM organic network interspersed with an α-HHG inorganic network, and there are more pores in the internal structure of the specimens than in the pure water group, with observable, incompletely reacted residual α-HHG grains. Figure 7e,f present FE-SEM images of the fracture section of the oven-dried specimens of CPO/PAM/α-HHG. The network within CPO/PAM/α-HHG is more homogeneous and dense than that within the PAM/α-HHG specimen, and no residual α-HHG grains are present. The flatness of its section is significantly improved, and the organic/inorganic network interpenetration structure is clearly visible. The results of the fracture observation corroborate the findings of the phase analysis in Section 2.3, indicating that the addition of CPO delays the hydration hardening process of CPO/PAM/α-HHG composites. The organic and inorganic networks are fully developed, enhancing the interspersal and fusion between the organic/inorganic networks, which leads to a flatter cross-section and significantly improves the mechanical strength of the composites.

### 2.5. Thermogravimetric Analysis of CPO/PAM/α-HHG Composite Materials

**Figure 8:**
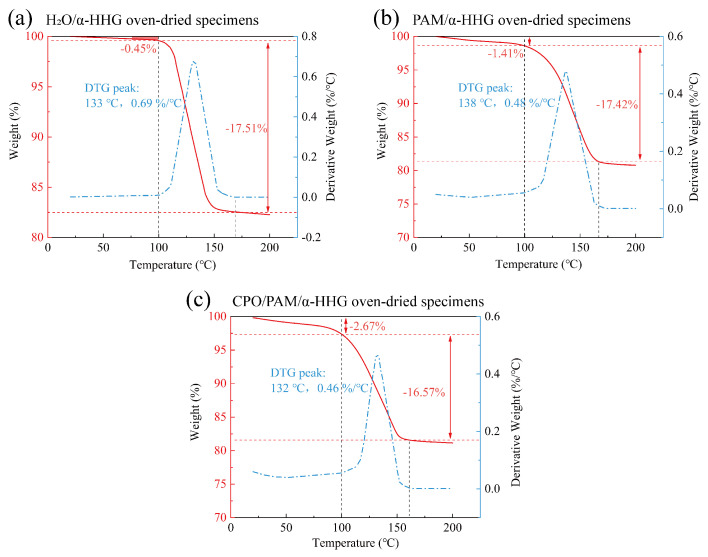
Thermogravimetric analysis (TGA) curves of the (**a**) oven-dried specimens of H_2_O/α-HHG, (**b**) oven-dried specimens of the PAM/α-HHG composite material, and (**c**) oven-dried specimens of the CPO/PAM/α-HHG composite material. The red curve illustrates the proportion of sample mass change with temperature, while the blue curve indicates the dependency of the rate of mass change on temperature, obtained through the differentiation of the TGA curve.

Figure 8 presents the thermogravimetric analysis (TGA) curves of the oven-dried samples of the pure water control group (standard consistency: 36 g of water per 100 g α-HHG), the PAM/α-HHG composite, and the CPO/PAM/α-HHG composite (CPO/PAM-L10). In these curves, the red line represents the variation in sample mass as a function of temperature, while the blue line indicates the corresponding rate of mass loss.

As shown in Figure 8a, the weight loss of the pure water control oven-dried specimens before 100 °C is the smallest at only 0.45%. A significant weight loss stage occurs between 100 and 167 °C, with a total weight reduction of 17.51%, corresponding to the loss of structural water in the α-HHG hydration–hardened body. As shown in Figure 8b, the weight loss of the oven-dried specimens of the PAM/α-HHG composites before 100 °C is 1.41%, a higher percentage than the 0.45% observed in the pure water group. This increase is attributed to the organic gel network within the specimen, which encapsulates a portion of the free water. The evaporation of this water results in a more significant weight loss than in the pure water group. As shown in Figure 8c, the weight loss of the oven-dried specimens of the CPO/PAM/α-HHG composites before 100 °C is 2.67%, which is 1.9 times that of the PAM/α-HHG composites at this stage. This phenomenon is likely due to the more complete development of the organic network in the CPO/PAM/α-HHG composites, which leads to a higher amount of free water confined within the gel. Consequently, this results in increased weight loss before 100 °C. This speculation is supported by comparing Figure 7d,e, which show that the pore sizes in the CPO/PAM/α-HHG composites are finer than those in the PAM/α-HHG composites. These pores are widely distributed within the matrix and are likely locations for confined free water. The denser organic network structure within CPO/PAM/α-HHG exhibits a greater ability to retain free water than PAM/α-HHG. This retained free water is continuously available for the hydration of α-HHG, which explains why no residual α-HHG grains are observed in the CPO/PAM/α-HHG composites.

### 2.6. Water Resistance Analysis

The wet compressive strength (R2) and water resistance coefficient (f) of the α-HHG pure water control, PAM/α-HHG composites, and CPO/PAM/α-HHG-L10 composites are tested, with the results presented in Table 1. The wet compressive strength (R2) of CPO/PAM/α-HHG-L10 is 14.03 MPa, with a softening coefficient of 0.20, which is 54.5% lower than that of the pure water control (0.44) and 5.0% lower than that of PAM/α-HHG (0.21). The reduction in the softening coefficient of the CPO/PAM/α-HHG-L10 composites is attributed to two main factors: First, the dry compressive strength of the CPO/PAM/α-HHG-L10 composites is significantly improved; i.e., the R1 value is substantially increased. Second, the CPO/PAM/α-HHG system, strengthened by CPO, features a denser organic network. This structure mitigates the water absorption and swelling-induced interfacial dislocation effect, thereby reducing the weakening of material strength and slightly increasing the R2 value [35], which is also corroborated by the thermogravimetric analysis results in Section 2.5.

Notably, the wet compressive strength (R2) of the CPO/PAM/α-HHG system is improved by 11.8% compared to that of the PAM/α-HHG system (12.55 MPa), despite a slight decrease in the softening coefficient. Furthermore, after water immersion followed by drying treatment, both composites successfully recover their original dry compressive strength, demonstrating their structural stability. This study demonstrates that the CPO modification effect broadens the practical engineering application scenarios for the composites while maintaining their water resistance.

### 2.7. Theoretical Investigation of Adsorption Behaviour Between PAM and CPO

**Figure 9:**
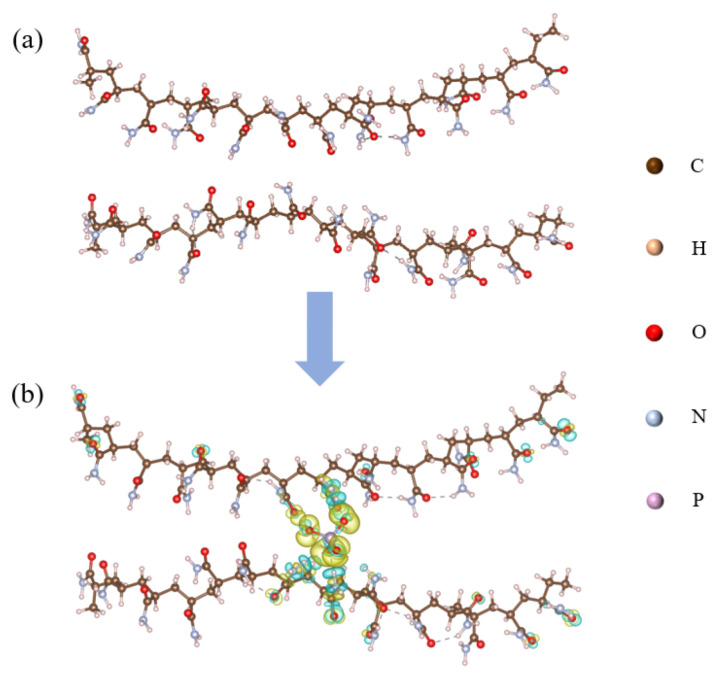
Schematic diagrams of PAM (**a**) and CPO/PAM (**b**). The isosurface in (**b**) is set to 0.01 e, with accumulation shown in yellow and depression shown in cyan.

To investigate the interaction between PAM and CPO, two individual long PAM chains (comprising 14 and 15 repeating units) were constructed without any direct connection between them, as shown in Figure 9a. An HPO_4_^2−^ ion was inserted between the two PAM chains to simulate the interaction between a single HPO_4_^2−^ ion and the two PAM chains, as illustrated in Figure 9b. Due to the limited solubility of CPO in solution and the subsequent hydrolysis of PO_4_^3−^ ions, leading to significantly higher concentrations of HPO_4_^2−^ and H_2_PO_4_^−^ than PO_4_^3−^, HPO_4_^2−^ was selected as the representative species for the calculations. The charge density difference results indicate that charge transfer occurred from the NH_2_ groups on the PAM chains towards the HPO_4_^2−^ ion, where the yellow regions represent charge accumulation, and the cyan regions represent charge depletion. A Bader charge analysis revealed that the HPO_4_^2−^ ion gained a total of 0.91 electrons from the surrounding NH_2_ groups on the PAM chains, suggesting that the addition of CPO significantly enhances the cross-linking interactions between PAM chains. Furthermore, the adsorption energy of the CPO/PAM model was calculated to be −4.36 eV using the following Equation (1), further confirming the stability of the connection between the PAM chains:

(1) $$E_{\mathrm{ads}}=E_{\mathrm{Total}}-E_{\mathrm{PAM}}-E_{\mathrm{HPO}}$$

## 3. Materials and Methods

### 3.1. Materials

The materials used were α-hemihydrate gypsum (CaSO_4_·0.5H_2_O, powder, Dinglijie Gypsum Powder Co., Ltd., Zaozhuang City, China); the standard water amount for every 100 g of gypsum is 36 g, with a specific surface area of 0.4 m^2^/g), AM (C_3_H_5_NO, Macklin Reagents Co., Ltd., Shanghai, China; analytical grade), ammonium persulphate (APS, (NH_4_)_2_S_2_O_8_, Macklin Reagents Co., Ltd., Shanghai, China; analytical grade), N,N’-methylenebisacrylamide (MBA, C_7_H_10_N_2_O_2_, Macklin Reagents Co., Ltd., Shanghai, China; analytical grade), tetramethylethylenediamine (TEMED, C_6_H_16_N_2_, Aladdin Reagents Co., Ltd., Shanghai, China; analytical grade), phosphoric acid (H_3_PO_4_, Sinopharm Chemical Reagent Co., Ltd., Shanghai, China; analytical grade), calcium chloride dihydrate (CaCl_2_·2H_2_O, Macklin Reagents Co., Ltd., Shanghai, China; analytical grade), TEA (C_6_H_15_N, Macklin Reagents Co., Ltd., Shanghai, China; analytical grade), and ethanol (C_2_H_6_O, Sinopharm Chemical Reagent Co., Ltd., Shanghai, China; analytical grade). Solutions were prepared with deionised water (Molecular Lab water ultra-purifier, Shanghai, China).

### 3.2. Preparation of TEA-Capped CPO

**Figure 10:**
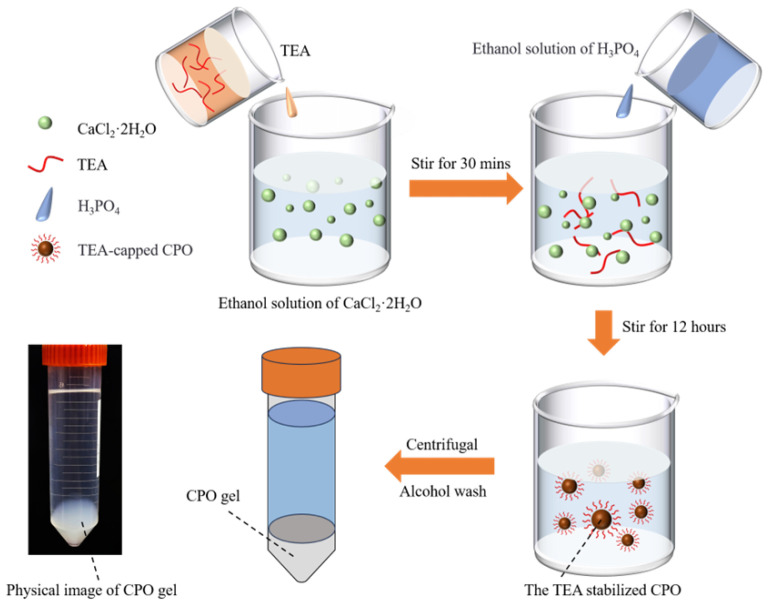
Schematic diagram of the preparation process for the CPO gel and a physical image of the CPO gel.

The preparation process of the CPO gel, along with a physical diagram, is illustrated in Figure 10. A total of 0.882 g of CaCl_2_·2H_2_O was dissolved in 300 mL of anhydrous ethanol, after which 16.635 mL of TEA was added. The mixture was continuously stirred using a magnetic stirrer at 600 rpm for 30 min at 25 °C. After pre-mixing 0.461 mL of phosphoric acid (80 wt%) with 15 mL of anhydrous ethanol, this mixture was added dropwise to the previous solution to facilitate the reaction. The reaction system was stirred continuously at room temperature (20 ± 3 °C) for 12 h, resulting in the formation of a white colloidal suspension. During this process, TEA capped the CPO, leading to the formation of inorganic CPO [14]. The suspension was centrifuged at 8000 rpm for 5 min, and the supernatant was then discarded. The precipitate was washed ultrasonically with 15 mL of ethanol and centrifuged twice to obtain a high-purity CPO gel. The CPO gel was subsequently dispersed in anhydrous ethanol and homogenised by sonication for 30 min to form a stable emulsion. Prior to use, the CPO gel was collected by centrifugation at 8000 rpm for 5 min.

### 3.3. Preparation of CPO/PAM Precursor Solutions

Table 2 presents the composition of the seven precursor liquids. A total of 8.8850 g of AM was weighed and added to 40 mL of deionised water, together with varying amounts of CPO gel, while stirring continuously at 500 rpm until the acrylamide was fully dissolved. Subsequently, 0.0285 g of ammonium persulphate (APS, as an initiator), 0.0193 g of methylenebisacrylamide (MBA, as a cross-linking agent), and 10 mL of N,N,N’,N’-tetramethylethylenediamine (TMEDA, as an accelerator) at a concentration of 0.0086 mol/L were added sequentially to the acrylamide solution. The mixture was then stirred continuously for 8 min until a homogeneous precursor solution was achieved.

### 3.4. Evaluation of the Drawable Time of CPO/PAM Composite Hydrogels

A CPO/PAM precursor solution was prepared at room temperature (20 ± 3 °C). A glass rod (5 mm in diameter) was inserted into the precursor solution and subsequently lifted to observe the resulting changes in the solution. Measurements were recorded every 30 s, with timing commencing when a string length exceeding 5 cm was observed. Timing continued until the string length was no longer able to reach 5 cm. The total elapsed time was recorded as the drawable time of the gel.

### 3.5. Preparation and Mechanical Property Testing of CPO/PAM/α-HHG Composites

**Figure 11:**
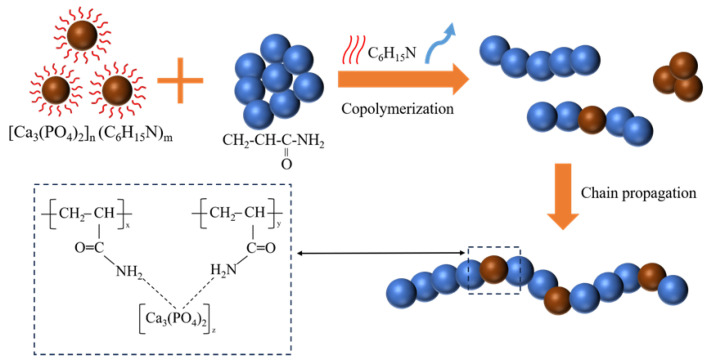
Schematic illustration of the copolymerisation process and the molecular chain structure of the homogeneous CPO/PAM.

Figure 11 presents a schematic representation of the polymerisation process of the CPO gel with AM. During this process, three distinct polymerisation reactions occur: first, the AM monomer undergoes free radical polymerisation; second, a small amount of CPO is polymerised through the evaporation-induced initiation by TEA; and, third, CPO and AM monomers are polymerised through hydrogen bonding. All organic, inorganic, and organic/inorganic oligomers undergo further polymerisation via chain growth, ultimately forming organic/inorganic copolymers.

**Figure 12:**
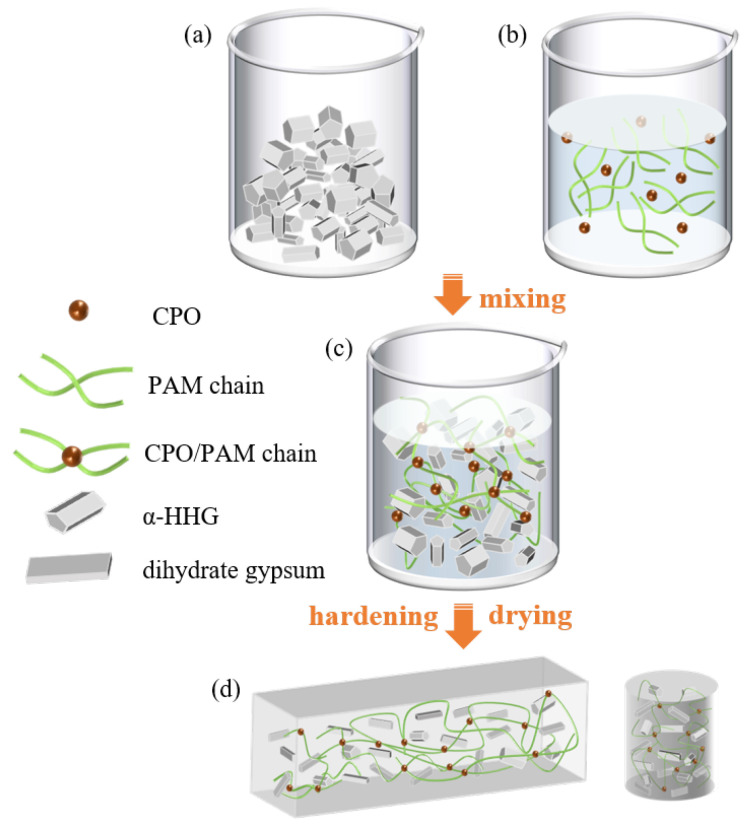
Schematic diagram of the preparation process for CPO/PAM/α-HHG composite samples. (**a**) α-HHG, (**b**) CPO/PAM precursor solution, (**c**) CPO/PAM/α-HHG composite slurry, (**d**) CPO/PAM/α-HHG composite oven-dried samples.

Figure 12 depicts the preparation process of the CPO/PAM/α-HHG composites. According to the formulation provided in Table 2 (Section 3.3), the freshly prepared CPO/PAM precursor solution was immediately combined with α-HHG at a specified ratio and thoroughly mixed to obtain a homogeneous composite slurry (Figure 12c). The slurry was then cast into moulds and allowed to hydrate and harden at ambient temperature (20 ± 3 °C). After hydration, the specimens were transferred to a drying oven set to 50 °C and dried until reaching a stable mass, producing the final oven-dried CPO/PAM/α-HHG composite samples (Figure 12d).

Following the GB/T (17669.3-1999) standard “Gypsum: Determination of Mechanical Properties” [36], mechanical property testing was conducted using a YWA-300C mechanical testing machine (Jinan Zhongluchang Testing Machine Manufacturing Co., Ltd., Jinan, China) (Figure S1). Flexural strength was measured on rectangular specimens (60×20×20 mm^3^) under a loading rate of 20 N/s, and compressive strength was determined using cylindrical specimens (Φ20×20 mm^2^) loaded at 2000 N/s. Each test was repeated three times for every sample, and the average value was reported as the final strength result.

### 3.6. Evaluation of Physical Properties of CPO/PAM/α-HHG Composite Slurry

In this study, the physical properties of the CPO/PAM/α-HHG composite slurry were evaluated using three indicators: temperature variation, initial setting time, and flowability. All tests were conducted following the GB/T (17669.5-1999) standard [37] for “Gypsum: Determination of Physical Properties of Powder”.

(1) Temperature variation: To prepare the composite slurry, the CPO/PAM precursor solution was mixed with 100 g of α-HHG to achieve standard consistency. Immediately after mixing, an automatic temperature sensor (DS18B20, Dallas Semiconductor, Dallas, TX, USA) was inserted into the slurry to continuously record the temperature evolution during hydration and hardening at 20 ± 3 °C.

(2) Initial setting time: The initial setting time was determined using a Vicat apparatus (ISO standard, Wuxi Zhongke Building Material Instrument Co., Ltd., Wuxi, China) (Figure S2). The composite slurry was poured into the annular mould of the Vicat apparatus. Timing began at the moment of mixing, and measurements were recorded every 15 s. The setting time was defined when the pointer penetration reached a specified limit.

(3) Flowability: The slurry’s flowability was assessed by measuring the spread diameter on a glass plate (Figure S3). The freshly prepared slurry was poured into a standard mould (LT-JJLD, Beijing Zhongke Luda Test Instrument Co., Ltd., Beijing, China) until level with the top surface. The mould was then swiftly lifted vertically, and the diameter of the spread was measured after 15 s to quantify the flowability.

### 3.7. Structural Analysis of CPO/PAM/α-HHG Composites

The oven-dried CPO/PAM/α-HHG composite specimens were finely ground and sieved through a 200-mesh screen. Subsequently, the powder samples were analysed for their structural characteristics by X-ray diffraction (XRD) using a PANalytical Empyrean diffractometer (Almelo, The Netherlands) with Cu K*α* radiation (λ = 0.154 nm). The 2θ range was scanned from 10° to 90° at a step size of 0.03°/min.

Surface morphology and fracture features were investigated by field-emission scanning electron microscopy (FE-SEM, SU8010, Hitachi, Tokyo, Japan) operating at an accelerating voltage of 5 kV. Prior to SEM observation, the fractured specimens were first freeze-dried for 12 h, followed by oven drying at 50 °C for an additional 12 h to ensure sample stability.

### 3.8. Thermogravimetric Analysis

The change in material mass with temperature was evaluated using a thermogravimetric analyser (TA Instruments, New Castle, DE, USA). The heating procedure commenced at room temperature and progressed to 200 °C at a rate of 10 °C/min, conducted under a protective atmosphere of argon.

### 3.9. Water Resistance Analysis

Six cylindrical specimens of CPO/PAM/α-HHG composites were prepared following the sample preparation method outlined in Section 3.5 and dried in an oven at 50 °C until they reached a constant weight. Three of the specimens were tested directly for compressive strength, and the calculated average value was recorded as the dry compressive strength (R1). The other three specimens were soaked in water for 24 h, removed, and subsequently tested for compressive strength; the average value was recorded as the wet compressive strength (R2). The softening coefficient of the composites (f) could be calculated from the ratio of R2 to R1, as indicated in Equation (2):

(2) f=R1/R2

### 3.10. Computational Details

The Vienna Ab-initio Simulation Package (VASP) [38,39,40] was employed for the calculations conducted in this study. The calculations were performed using the Perdew–Burke–Ernzerhof (PBE) functional [41] within the generalised gradient approximation (GGA) framework. A plane-wave cutoff energy of 450 eV was applied, and the convergence criterion for electronic iterations was set to an energy difference of less than 10^−6^ eV between two successive steps. Structural relaxation was continued until the residual forces on all atoms were reduced to below 0.1 eV/Å. Charge density difference visualisations were carried out using the VESTA software package [42].

## 4. Conclusions

This study synthesises a novel CPO/PAM/α-HHG composite material by polymerising a stable inorganic CPO precursor, end-capped with TEA, with an organic PAM hydrogel to form a CPO/PAM precursor solution. Subsequently, this precursor solution is mixed with inorganic α-HHG. The introduction of CPO allows both the gelation process and the α-HHG hydration reaction process of PAM to proceed sufficiently, resulting in the full development of both organic and inorganic networks, ultimately leading to the formation of an interspersed inorganic/organic dual-network structure.

When 37 g of CPO/PAM-L10 precursor liquid is added to each 100 g of α-HHG, the initial solidification time of the CPO/PAM/α-HHG composites is 26.08 min, which is slightly prolonged by about 4.0% compared to that of the PAM/α-HHG composites; additionally, the flexural and compressive strengths of the oven-dried specimens are 27.59 MPa and 68.48 MPa, which are 38.8% and 14.1% higher than those of the PAM/α-HHG composites, respectively. The mechanical properties of the CPO/PAM/α-HHG composites reach their optimum when the addition amount of CPO is 0.1 g.

The enhancement of the mechanical strength, the improvement in the water resistance performance, and the prolongation of the initial setting time significantly broaden the application prospects of the composites in practical engineering. However, experimental investigations on the weathering resistance, corrosion resistance, and high-temperature stability of the composite material are still lacking, and further studies in these areas are warranted in the future.

## Figures and Tables

**Table 1:** Softening coefficient and wet compressive strength table of H_2_O/α-HHG, the PAM/α-HHG composite material, and the CPO/PAM/α-HHG-L10 composite material.

	H_2_O/α-HHG	PAM/α-HHG	CPO/PAM/α-HHG-L10
Softening Coefficient	0.44	0.21	0.20
Wet Compressive Strength (MPa)	15.46	12.55	14.03

**Table 2:** Composition of seven different precursor solutions.

Precursor Solutions	AM (g)	APS (g)	MBA (g)	TEMED (mL)	Deionised Water (mL)	CPO (g)
PAM-L	8.8850	0.0285	0.0193	0.0645	50	0
CPO/PAM-L2.5	8.8850	0.0285	0.0193	0.0645	50	0.0250
CPO/PAM-L5	8.8850	0.0285	0.0193	0.0645	50	0.0500
CPO/PAM-L7.5	8.8850	0.0285	0.0193	0.0645	50	0.0750
CPO/PAM-L10	8.8850	0.0285	0.0193	0.0645	50	0.1000
CPO/PAM-L12.5	8.8850	0.0285	0.0193	0.0645	50	0.1250
CPO/PAM-L15	8.8850	0.0285	0.0193	0.0645	50	0.1500

## Data Availability

The original contributions presented in this study are included in the article. Further inquiries can be directed to the corresponding author(s).

## Supplementary Materials

The following supporting information can be downloaded at: https://www.mdpi.com/article/10.3390/molecules30092002/s1, Figure S1: Schematic diagram of the mechanical properties test of the CPO/PAM/α-HHG composites; Figure S2: Schematic diagram of the initial setting time of the CPO/PAM/α-HHG composite slurry; Figure S3: Schematic diagram of the flowability of the CPO/PAM/α-HHG composite slurry; Table S1: The initial setting time of the α-HHG-based composites under varying addition amounts of different precursor solutions; Table S2: The flexural strength of the α-HHG-based composites under varying addition amounts of different precursor solutions; Table S3: The compressive strength of the α-HHG-based composites under varying addition amounts of different precursor solutions; Table S4: The initial setting time, flexural strength, and compressive strength of the CPO/PAM/α-HHG composites with varying CPO addition amounts (the addition amount of the precursor solution was set to 37 g).

## References

[1] Ye Y., Huang Q., Li X. (2024). Effect of Fiber Loading on Mechanical and Flame-Retardant Properties of Poplar-Fiber-Reinforced Gypsum Composites. Molecules.

[2] Guo G., Gao G., Jiang W., Wang X., Pei M., Wang L. (2023). Synthesis of Triblock Polycarboxylate Superplasticizers with Well-Defined Structure and Its Dispersing Performance in *β*-Hemihydrate Gypsum. Molecules.

[3] Xie S., Ji Z., Yang Y., Hou G., Wang J. (2016). Electromagnetic wave absorption enhancement of carbon black/gypsum based composites filled with expanded perlite. Compos. Part B Eng..

[4] Xie Z., Liu X., Zhang Z., Wei C., Gu J. (2024). Application of the Industrial Byproduct Gypsum in Building Materials: A Review. Materials.

[5] Ravenhill E.R., Kirkman P.M., Unwin P.R. (2016). Microscopic Studies of Calcium Sulfate Crystallization and Transformation at Aqueous-Organic Interfaces. Cryst. Growth Des..

[6] Vidales-Barriguete A., Santa-Cruz-Astorqui J., Pina-Ramirez C., Kosior-Kazberuk M., Kalinowska-Wichrowska K., Atanes-Sanchez E. (2021). Study of the Mechanical and Physical Behavior of Gypsum Boards with Plastic Cable Waste Aggregates and Their Application to Construction Panels. Materials.

[7] Tang Y., Gao J., Liu C., Chen X., Zhao Y. (2019). Dehydration Pathways of Gypsum and the Rehydration Mechanism of Soluble Anhydrite *γ*-CaSO_4_. ACS Omega.

[8] Xie Y., Yang L., Wang C., Feng X., Tang Z., Liu Z. (2023). Production and properties of the polyvinyl alcohol modified macro-defect-free alpha-hemihydrate gypsum composite. Constr. Build. Mater..

[9] Triantafyllou E., Karydis-Messinis A., Moschovas D., Kyriakaki C., Vasilopoulos K.C., Giannakas A.E., Karakassides M.A., Avgeropoulos A., Zafeiropoulos N.E., Salmas C.E. (2024). Microwave-Assisted Extraction of Cellulose from Aloe Vera Plant Residue and Preparation of Cellulose Nanocrystal-Poly(vinyl alcohol) Hydrogels. Molecules.

[10] Schexnailder P., Schmidt G. (2009). Nanocomposite polymer hydrogels. Colloid Polym. Sci..

[11] El Sayed M.M. (2023). Production of Polymer Hydrogel Composites and Their Applications. J. Polym. Environ..

[12] Song X., Lu G., Wang J., Zheng J., Sui S., Li Q., Zhang Y. (2022). Molecular Dynamics-Assisted Design of High Temperature-Resistant Polyacrylamide/Poloxamer Interpenetrating Network Hydrogels. Molecules.

[13] Ho T.C., Chang C.C., Chan H.P., Chung T.W., Shu C.W., Chuang K.P., Duh T.H., Yang M.H., Tyan Y.C. (2022). Hydrogels: Properties and Applications in Biomedicine. Molecules.

[14] Koh J.K., Lai C.W., Johan M.R., Gan S.S., Chua W.W. (2022). Recent advances of modified polyacrylamide in drilling technology. J. Pet. Sci. Eng..

[15] Zhao Y., Zhou J., Xu X., Liu W., Zhang J., Fan M., Wang J. (2009). Synthesis and characterization of a series of modified polyacrylamide. Colloid Polym. Sci..

[16] Ma T., Wu Y., Liu N., Wu Y. (2020). Hydrolyzed polyacrylamide modified diatomite waste as a novel adsorbent for organic dye removal: Adsorption performance and mechanism studies. Polyhedron.

[17] Kaşgöz H., Özgümüş S., Orbay M. (2003). Modified polyacrylamide hydrogels and their application in removal of heavy metal ions. Polymer.

[18] Zheng X., Zheng H., Xiong Z., Zhao R., Liu Y., Zhao C., Zheng C. (2020). Novel anionic polyacrylamide-modify-chitosan magnetic composite nanoparticles with excellent adsorption capacity for cationic dyes and pH-independent adsorption capability for metal ions. Chem. Eng. J..

[19] Yu Y., Mu Z., Jin B., Liu Z., Tang R. (2020). Organic-Inorganic Copolymerization for a Homogenous Composite without an Interphase Boundary. Angew. Chem. Int. Ed..

[20] Sophia M., Sakthieswaran N. (2019). Synergistic effect of mineral admixture and bio-carbonate fillers on the physico-mechanical properties of gypsum plaster. Constr. Build. Mater..

[21] Dima C., Badanoiu A., Cirstea S., Nicoara A.I., Stoleriu S. (2020). Lightweight Gypsum Materials with Potential Use for Thermal Insulations. Materials.

[22] Zha F., Qiao B., Kang B., Xu L., Chu C., Yang C. (2021). Engineering properties of expansive soil stabilized by physically amended titanium gypsum. Constr. Build. Mater..

[23] Ding X., Wang S., Dai R., Chen H., Shan Z. (2022). Hydrogel beads derived from chrome leather scraps for the preparation of lightweight gypsum. Environ. Technol. Innov..

[24] Wu Q., Zhu Z., Li S., Wang S., Chen B. (2017). Effect of polyacrylic ester emulsion on mechanical properties of macro-defect free desulphurization gypsum plaster. Constr. Build. Mater..

[25] Jia C., Wu L., Chen Q., Ke P., De Yoreo J.J., Guan B. (2020). Structural evolution of amorphous calcium sulfate nanoparticles into crystalline gypsum phase. CrystEngComm.

[26] Charai M., Mghazli M.O., Channouf S., El Hammouti A., Jagadesh P., Moga L., Mezrhab A. (2023). Lightweight waste-based gypsum composites for building temperature and moisture control using coal fly ash and plant fibers. Constr. Build. Mater..

[27] Pedrajas D.L., Franco M.C., Saenz I.G., Mellado F.J.R., Romero J.F.R., Simon A.M.B. (2022). Polystyrene nanoparticles slurry as an additive for developing insulating and waterproof gypsum composites. Appl. Therm. Eng..

[28] Mroz P., Mucha M. (2018). Hydroxyethyl methyl cellulose as a modifier of gypsum properties. J. Therm. Anal. Calorim..

[29] Thompson B.R., Horozov T.S., Stoyanov S.D., Paunov V.N. (2018). Hierarchically porous composites fabricated by hydrogel templating and viscous trapping techniques. Mater. Des..

[30] Chen Y., Mi Z., Yang J., Zheng X., Wang H., Record M.C., Boulet P., Wang J., Albina J.M., Huang Y. (2024). Synthesis and Characterisation of Hemihydrate Gypsum-Polyacrylamide Composite: A Novel Inorganic/Organic Cementitious Material. Materials.

[31] Zhao H., Hu G.H., Ye G.B., Ren X.M., Zhang Q.C., Jiang T. (2018). Effects of superplasticisers on hydration process, structure and properties of *α*-hemihydrate calcium sulfate. Adv. Cem. Res..

[32] Guan B., Ye Q., Zhang J., Lou W., Wu Z. (2010). Interaction between *α*-calcium sulfate hemihydrate and superplasticizer from the point of adsorption characteristics, hydration and hardening process. Cem. Concr. Res..

[33] Beaugnon F., Preturlan J.G.D., Fusseis F., Gouillart E., Quiligotti S., Wallez G. (2022). From atom level to macroscopic scale: Structural mechanism of gypsum dehydration. Solid State Sci..

[34] Kyere-Yeboah K., Qiao X.c. (2024). Application of cold plasma technology for the simultaneous degradation and viscosity removal of polyacrylamide and its copolymers from contaminated wastewater. J. Environ. Manag..

[35] Gong J.P., Katsuyama Y., Kurokawa T., Osada Y. (2003). Double-network hydrogels with extremely high mechanical strength. Adv. Mater..

[36] (1999). Gypsum Plasters—Determination of Mechanical Properties.

[37] (1999). Gypsum Plasters—Determination of Physical Properties of Powder.

[38] Kresse G., Hafner J. (1993). Ab Initio Molecular Dynamics for Liquid Metals. Phys. Rev. B.

[39] Kresse G., Furthmüller J. (1996). Efficiency of Ab-Initio Total Energy Calculations for Metals and Semiconductors Using a Plane-Wave Basis Set. Comput. Mater. Sci..

[40] Dudarev S.L., Botton G.A., Savrasov S.Y., Humphreys C.J., Sutton A.P. (1998). Electron-Energy-Loss Spectra and the Structural Stability of Nickel Oxide: An LSDA+U Study. Phys. Rev. B.

[41] Perdew J.P., Burke K., Ernzerhof M. (1996). Generalized Gradient Approximation Made Simple. Phys. Rev. Lett..

[42] Momma K., Izumi F. (2011). VESTA3 for three-dimensional visualization of crystal, volumetric and morphology data. J. Appl. Crystallogr..

