# Supplementary material for: Triethylamine-Capped Calcium Phosphate Oligomers/Polyacrylamide Synergistically Reinforced α-Hemihydrate Gypsum Composites: A Mechanistic Study on Mechanical Strengthening via Organic/Inorganic Interpenetrating Networks
Source: Molecules. 2025 Apr 30;30(9):2002. doi: 10.3390/molecules30092002 (PMC12073873; doi:10.3390/molecules30092002)
Supplement: Supplementary file 1 [file molecules-30-02002-s001.zip › molecules-3570855-supplementary.pdf]

Supplemental Information

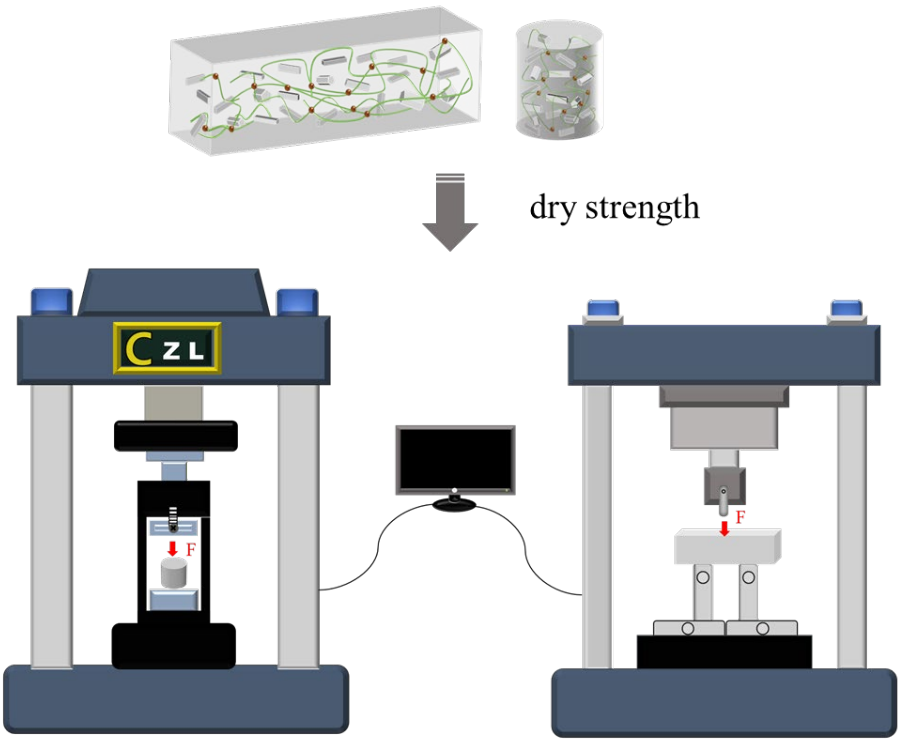

Figure S1 Schematic diagram of mechanical properties test of CPO/PAM/α-HHG composites

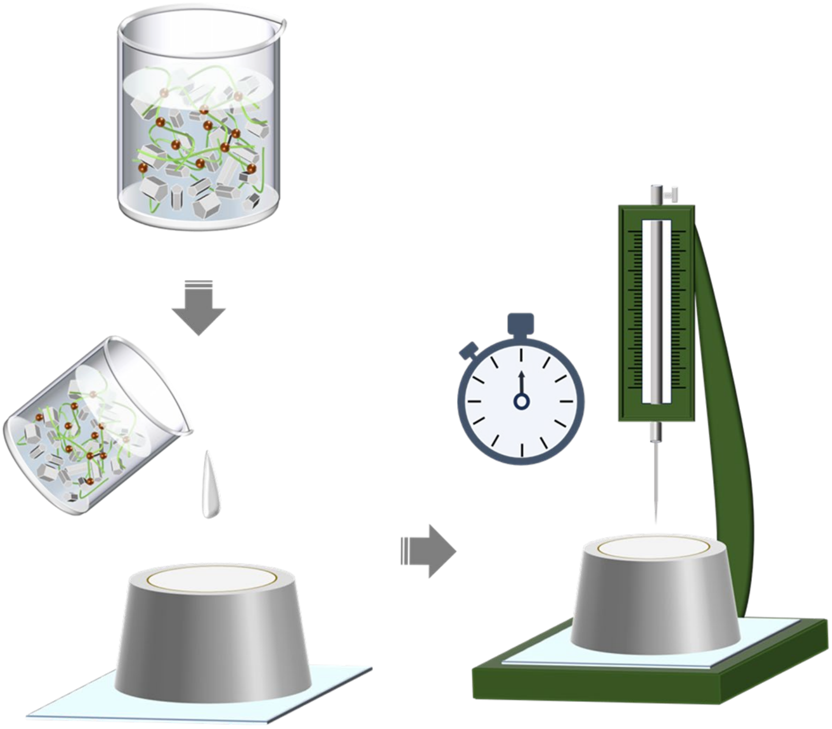

Figure S2 Schematic diagram of the The initial setting time of CPO/PAM/α-HHG composite slurry

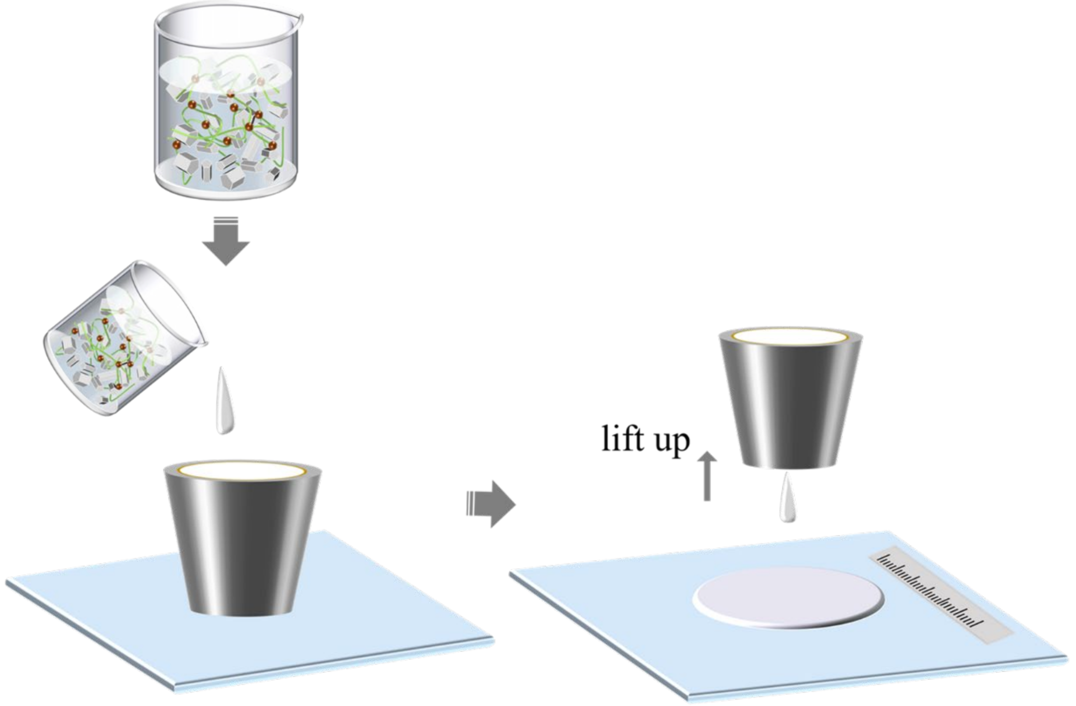

Figure S3 Schematic diagram of the flowability of the CPO/PAM/α-HHG composite slurry

Table S1 Initial setting time of α-HHG-based composites under varying addition amounts of different precursor solutions

Addition of the solution (g)	33	34	35	36	37
H_2O	10 min	10.8 min	11.1 min	11.1 min	11.8 min
PAM-L	22.1 min	23.5 min	23.8 min	24.4 min	25.7 min
CPO/PAM-L10	22.9 min	24.1 min	24.7 min	25.2 min	26.7 min

Table S2 Flexural strength of α-HHG-based composites under varying addition amounts of different precursor solutions

Addition of the solution (g)	35	36	37
H_2O	15.79 MPa	15.68 MPa	15.55 MPa
PAM-L	20.11 MPa	19.02 MPa	19.85 MPa
CPO/PAM-L10	28.13 MPa	19.85 MPa	27.56 MPa

Table S3 Compressive strength of α-HHG-based composites under varying addition amounts of different precursor solutions

Addition of the solution (g)	35	36	37
H_2O	32.18 MPa	35.01 MPa	33.90 MPa
PAM-L	61.03 MPa	60.47 MPa	59.98 MPa
CPO/PAM-L10	68.65 MPa	68.73 MPa	68.48 MPa

Table S4 Initial setting time, flexural strength, and compressive strength of CPO/PAM/α-HHG composites with varying CPO addition(The addition amount of the precursor solution was set to 37 g)

Addition of the CPO (g)	-	0.025	0.05	0.075	0.1	0.125	0.15
Initial setting time (min)	25.1	25.15	25.35	25.7	26.1	26.3	26.55
Flexural strength (MPa)	19.85	23.88	25.32	26.76	27.56	27.29	26.56
Compressive strength (MPa)	60.06	62.52	64.92	66.98	68.48	68.07	66.63
